# Supplementary figures and images for: Gut microbiota influences onset of foraging-related behavior but not physiological hallmarks of division of labor in honeybees
Source: mBio. 2024 Jul 29;15(9):e01034-24. doi: 10.1128/mbio.01034-24 (PMC11389387; doi:10.1128/mbio.01034-24)

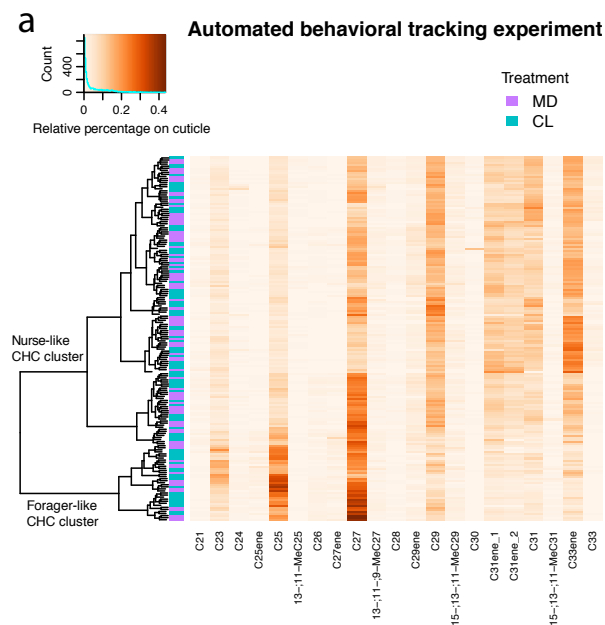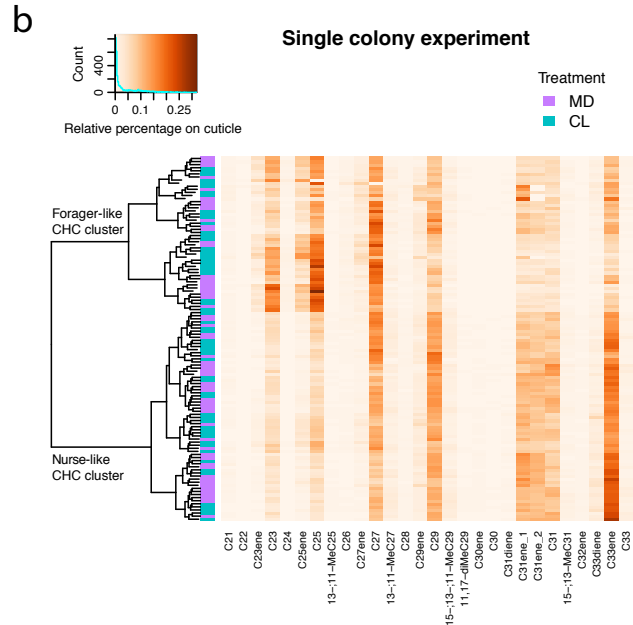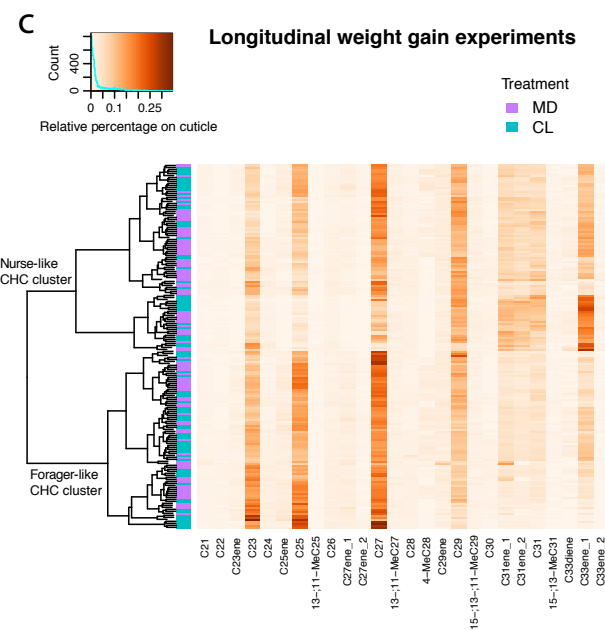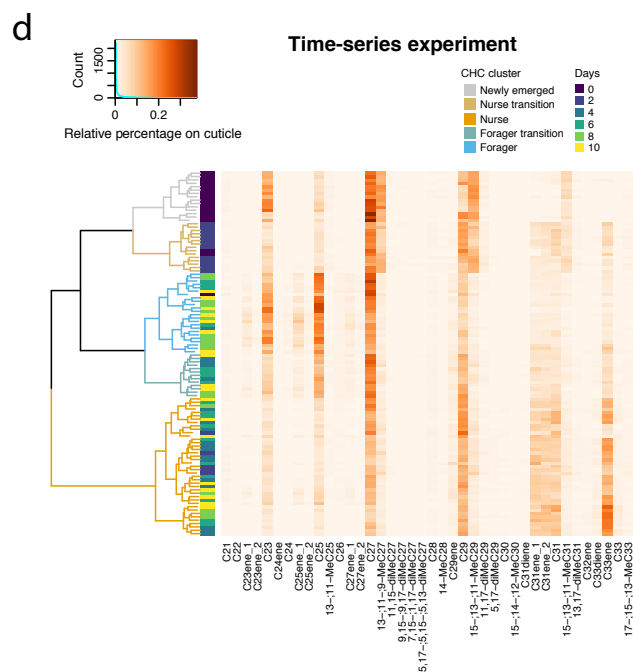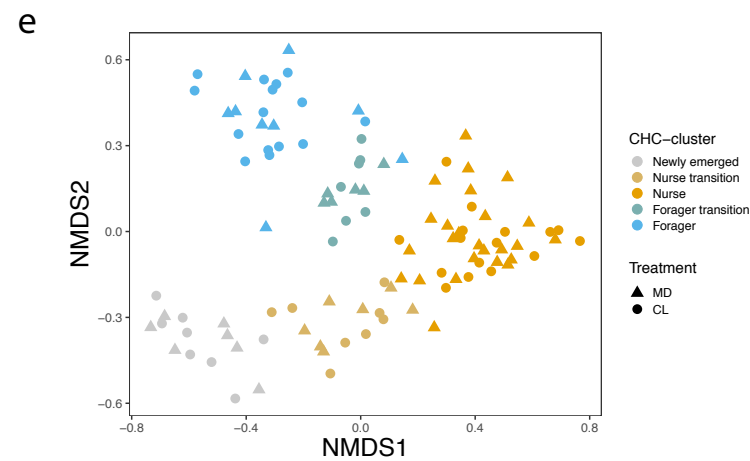

Supplement: Figure S1 — Heatmaps of relative abundance of detected CHCs on the cuticle of gnotobiotic bees. [file mbio.01034-24-s0001.pdf]

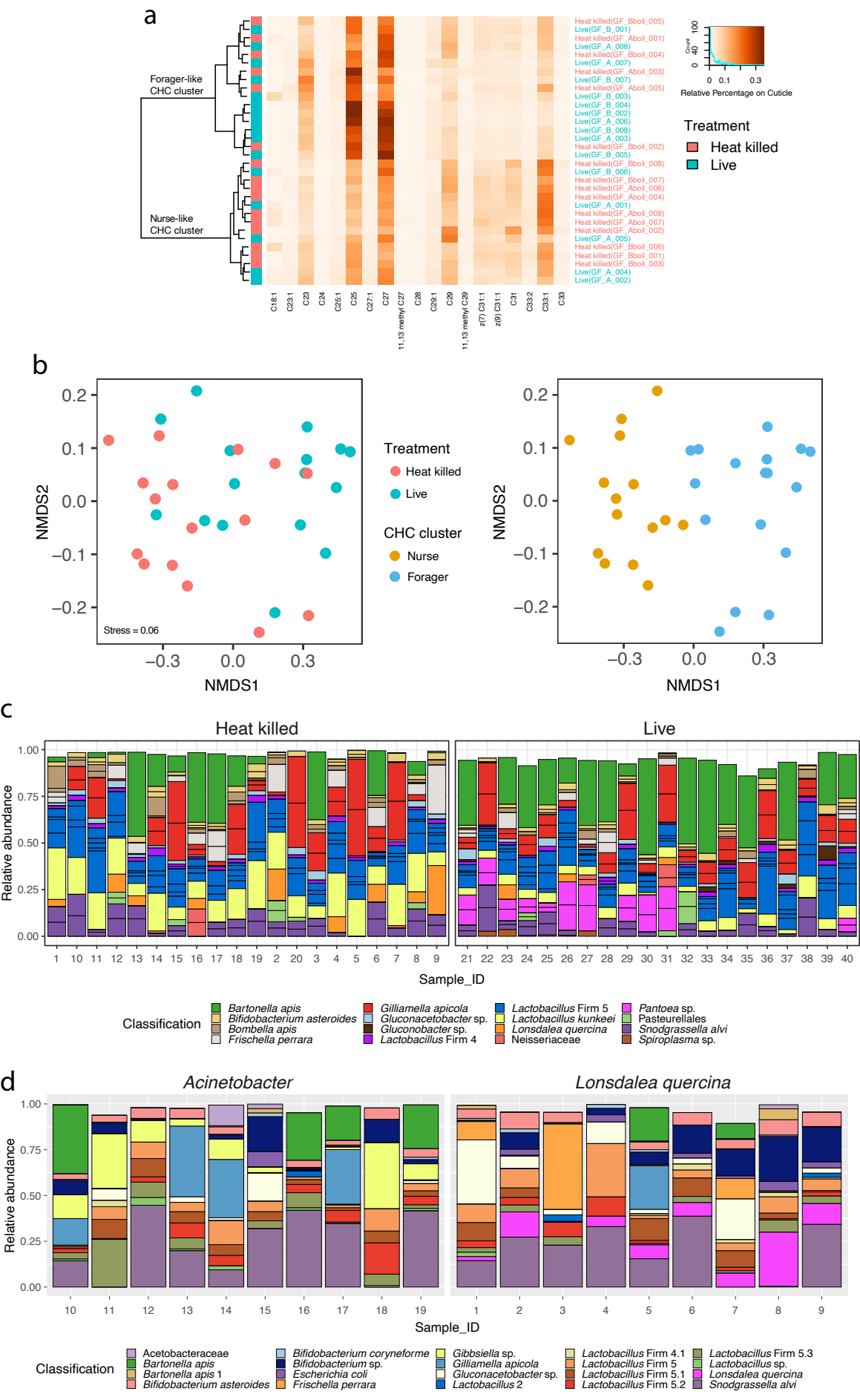

Supplement: Figure S2 — Re-analyses of two experiments in Vernier et al. (33). [file mbio.01034-24-s0002.pdf]
