# Supplementary material for: Gut microbiota influences onset of foraging-related behavior but not physiological hallmarks of division of labor in honeybees
Source: mBio. 2024 Jul 29;15(9):e01034-24. doi: 10.1128/mbio.01034-24 (PMC11389387; doi:10.1128/mbio.01034-24)
Supplement: Supplemental text — Captions for supplemental files. [file mbio.01034-24-s0003.docx]

**Supplementary Information**

**Supplementary Figure 1.** Heatmaps of relative abundance of detected CHCs on the cuticle of gnotobiotic bees in the automated behavioral tracking experiment (a), single colony CHC experiment (b), weight gain experiments (c) and time-series CHC experiment (d). The dendrograms towards the left show clustering of CHC profiles based on Euclidean distances using Ward's criterion. (e) Non-metric multidimensional scaling (NMDS) of Bray-Curtis dissimilarities between CHC profiles in the time-series CHC experiment, where color represents the CHC clusters identified in the dendrogram in panel (d) and shapes indicate the gut microbiota treatment groups.

**Supplementary Figure 2.** Re-analyses of two experiments in Vernier *et al*. (33). (a) Heatmap of relative abundances of detected CHCs in the live vs. heat-killed inoculum experiment (Figure 2E in Vernier *et al*. (33)). (b) Non-metric multidimensional scaling (NMDS) of Bray-Curtis dissimilarities between CHC profiles in the live vs. heat-killed inoculum experiment, with samples colored either by gut microbiota treatment group or by CHC clusters identified in panel (a). (c) Stacked bars showing the relative abundance of different amplicon sequence variants (ASVs) in the live vs. heat-killed inoculum experiment. (d) Stacked bars showing the relative abundance of different ASVs in the mono-inoculation experiment (Figure 3B in Vernier *et al*. (33)). Sub-bars of the same color show distinct ASVs with the same classification. For ease of visualization, the stacked bars show only ASVs that had a minimum of 2% relative abundance in two samples.

**Supplementary Table 1.** Median relative percentages with median absolute deviation (MAD) of all cuticular hydrocarbons identified in each experiment.

**Supplementary Table 2.** Results of differential gene expression analyses of brain and gut samples between CHC-classified nurses and foragers in the RNA-sequencing experiment, reported in separate sheets for each pair-wise comparison.

**Supplementary Table 3.** ASVs that had an FDR-corrected *P*<0.05 in DESeq2 analyses of differential relative abundance between the live and heat-killed treatments reported in Figure 2D in Vernier *et al*. (33).

**Supplementary Table 4.** ASVs that had an FDR-corrected *P*<0.05 in DESeq2 analyses of differential relative abundance between the mono-inoculation treatments reported in Figure 3B in Vernier *et al*. (33).

**Supplementary Movie 1.** Example of the experimental boxes hosted under the automated behavioral tracking systems. Bees tended to behave differently in the nest and foraging arenas, with bees frequently attempting to fly once they entered the foraging arena.
