# Supplementary material for: Gut microbiota influences onset of foraging-related behavior but not physiological hallmarks of division of labor in honeybees
Source: mBio. 2024 Jul 29;15(9):e01034-24. doi: 10.1128/mbio.01034-24 (PMC11389387; doi:10.1128/mbio.01034-24)
Supplement: Table S3 — ASVs that had an FDR-corrected P<0.05 in DESeq2 analyses of differential relative abundance between the live and heat-killed treatments reported in Fig. 2D in Vernier et al. (33). [file mbio.01034-24-s0006.docx]

| **Supplementary Table 3.** ASVs that had an FDR-corrected *P*<0.05 in DESeq2 analyses of differential relative abundance between the live and heat-killed treatments reported in Figure 2D in Vernier *et al*. (32). | | | | | | |
| --- | --- | --- | --- | --- | --- | --- |
|  |  |  |  |  |  |  |
| **ASV** | **baseMean** | **log2FoldChange** | **lfcSE** | **stat** | **pvalue** | **padj** |
| *Lactobacillus kunkeei* | 197.4642872 | -1.625307855 | 0.269601997 | -6.028545304 | 1.65E-09 | 9.89E-08 |
| *Bombella apis* 1 | 19.04869298 | -7.720344454 | 1.292960849 | -5.971058179 | 2.36E-09 | 9.89E-08 |
| *Pantoea* sp. | 35.80501722 | 8.578730365 | 1.435100446 | 5.977790886 | 2.26E-09 | 9.89E-08 |
| *Pantoea* sp. 1 | 34.76508898 | 8.536205103 | 1.430723628 | 5.966355023 | 2.43E-09 | 9.89E-08 |
| *Gluconobacter* sp. | 8.477483677 | 6.499770841 | 1.677775273 | 3.874041385 | 0.000107045 | 0.003489674 |
