# Supplementary material for: Gut microbiota influences onset of foraging-related behavior but not physiological hallmarks of division of labor in honeybees
Source: mBio. 2024 Jul 29;15(9):e01034-24. doi: 10.1128/mbio.01034-24 (PMC11389387; doi:10.1128/mbio.01034-24)
Supplement: Table S4 — ASVs that had an FDR-corrected P<0.05 in DESeq2 analyses of differential relative abundance between mono-inoculation treatments reported in Fig. 3B in Vernier et al. (33). [file mbio.01034-24-s0007.docx]

| **Supplementary Table 4.** ASVs that had an FDR-corrected *P*<0.05 in DESeq2 analyses of differential relative abundance between mono-inoculation treatments reported in Figure 3B in Vernier *et al*. (32). | | | | | | |
| --- | --- | --- | --- | --- | --- | --- |
|  |  |  |  |  |  |  |
| **ASV** | **baseMean** | **log2FoldChange** | **lfcSE** | **stat** | **pvalue** | **padj** |
| *Gibbsiella* sp. | 76.02791 | -6.731117 | 1.053231 | -6.390924 | 1.65E-10 | 2.64E-09 |
| *Lonsdalea quercina* | 46.80626 | 8.878397 | 1.143415 | 7.764809 | 8.18E-15 | 2.62E-13 |
